# Thyroid or Thyreoid

**Published:** 1896-09

**Authors:** 


### THYROID OR THYREOID.

Since the introduction into therapy, and hence into popularity, of this gland, the study of it has extended even to the etymology of the word, and the addition of the "e" has become very general. From the derivation of the word from the Greek θυρεος, an oblong shield, and ειδος, like, it is difficult to understand how the spelling in the past should have entirely ignored the epsilon when it occurs in both the words from which the name of the gland is compounded. Thyreoid is entirely correct, thyroid being simply slovenliness.

